# Effects of PM Exposure on the Methylation of Clock Genes in A Population of Subjects with Overweight or Obesity

**DOI:** 10.3390/ijerph18031122

**Published:** 2021-01-27

**Authors:** Paola Monti, Simona Iodice, Letizia Tarantini, Francesca Sacchi, Luca Ferrari, Massimiliano Ruscica, Massimiliano Buoli, Luisella Vigna, Angela Cecilia Pesatori, Valentina Bollati

**Affiliations:** 1 Department of Preventive Medicine, Fondazione IRCCS Cà Granda Ospedale Maggiore Policlinico, 20122 Milan, Italy; paola.monti2@studenti.unimi.it (P.M.); luisella.vigna@policlinico.mi.it (L.V.); angela.pesatori@unimi.it (A.C.P.); 2 EPIGET—Epidemiology, Epigenetics and Toxicology Lab, Department of Clinical Sciences and Community Health, Università degli Studi di Milano, 20122 Milan, Italy; simona.iodice@unimi.it (S.I.); letizia.tarantini@unimi.it (L.T.); franci.sacchi@fastwebnet.it (F.S.); luca.ferrari@unimi.it (L.F.); 3 Department of Pharmacological and Biomolecular Sciences, Università degli Studi di Milano, 20133 Milan, Italy; massimiliano.ruscica@unimi.it; 4 Department of Pathophysiology and Transplantation, Università degli Studi di Milano, 20122 Milan, Italy; massimiliano.buoli@unimi.it; 5 Department of Neurosciences and Mental Health, Fondazione IRCCS Cà Granda Ospedale Maggiore Policlinico, 20122 Milan, Italy; 6 Center of Obesity and Work EASO Collaborating Centers for Obesity Management, 20122 Milan, Italy

**Keywords:** obesity, particulate matter, DNA methylation, clock genes, *CLOCK*, *PER*, *ARNTL*, *CRY*, circadian rhythms

## Abstract

The expression of clock genes, regulating the synchronization of metabolic and behavioral processes with environmental light/dark cycles, is regulated by methylation and might be influenced by short-term exposure to airborne particulate matter (PM), especially in individuals that are hypersensitive to proinflammatory cues. The present study aimed to evaluate the effects of PM_2.5_ and PM_10_ on the methylation profile of the clock genes *ARNTL*, *CLOCK*, *CRY1*, *CRY2*, *PER1*, *PER2*, and *PER3* in a population of 200 women with obesity. A significant association between PM_10_ exposure and the methylation of clock genes was found, namely, this was negative for *PER2* gene and positive for the *CLOCK*, *CRY1*, *CRY2*, and *PER3* genes. PM_2.5_ was negatively associated with methylation of *PER2* gene and positively with methylation of *CRY2* gene. Evidence was observed for effect modification from body mass index (BMI) regarding the *PER1* gene: as PM_2.5/10_ increases, DNA methylation increases significantly for relatively low BMI values (BMI = 25), while it decreases in participants with severe obesity (BMI = 51). PM may therefore alter the epigenetic regulation of clock genes, possibly affecting circadian rhythms. Future studies are needed to clarify how alterations in clock gene methylation are predictive of disease development and how obesity can modulate the adverse health effects of PM.

## 1. Introduction

Nowadays, bad air quality is considered one of the major concerns for public health. Exposure to excessive concentrations of air pollutants has been implicated in the etiology and/or worsening of several pathological conditions, ranging from cardiovascular and neurological diseases to cancer [1,2,3]. It is now generally recognized that one of the main pathological mechanisms elicited by airborne pollutants, including particulate matter (PM), is based on the activation of a systemic inflammatory cascade [4,5]. After inhalation, fine PM deposits throughout the respiratory tract and damages the airway epithelium. This process promotes the recruitment of innate immune cells and the release of proinflammatory mediators, including cytokines and chemokines [6,7].

Obesity is an important susceptibility factor to the noxious effects started by PM exposure since this condition is characterized per se by a state of chronic low-grade inflammation and increased systemic oxidative stress [3,8,9]. Indeed, the excessive visceral fat accumulation that characterizes subjects with obesity, or even overweight, perturbs the metabolic homeostasis of adipocytes, leading to a large secretion of adipokines, proinflammatory factors that stimulate the production of oxygen free radicals by leukocytes [10,11,12]. Moreover, it has been hypothesized that the daily-inhaled air volume by subjects with obesity is greater than would be expected for normal-weight individuals, thus potentially resulting in an increased uptake of airborne PM [13,14]. Therefore, particulate air pollution is likely to act as an external proinflammatory trigger that exacerbates the pre-existing inflammatory status of subjects with obesity, making them hypersensitive to PM exposure.

Recently, it has been suggested that PM_10_, one of the principal components of particulate air pollution, could influence the methylation of the genes that regulate the circadian cycle [15,16]. Circadian rhythms are given by biochemical and behavioral oscillations that occur in about 24 h, in a coordinated way with external light/dark cycles. For the proper regulation of such daily fluctuations, both central and peripheral clocks are required. The hypothalamic suprachiasmatic nucleus (SCN), whose neurons receive the light stimulus directly from the retina, poses as the main circadian pacemaker that sends the signal to the peripheral clocks (gastrointestinal tract, adipose tissue, liver, muscles, heart, lungs, adrenal glands) and synchronizes the rhythmicity of all the cells in the body [17]. At the molecular level, clock genes are responsible for the maintenance of the daily oscillations that characterize many aspects of the cellular metabolism and program several physiological and behavioral processes, including the sleep/wake cycle, body temperature, hormonal secretion, locomotor activity, and eating behavior [18]. Due to their fundamental role in cellular physiology and metabolism, the expression of clock genes must be tightly regulated to avoid circadian misalignments. The cellular rhythmicity is given by a complex regulatory pathway, known as transcriptional and translational feedback loop (TTFL), which consists of positive and negative interconnected circuits. In the positive arm, the transcriptional activators CLOCK and ARNTL/BMAL1 form a heterodimer that binds to the Enhancer box (E-box) within the promoter region of genes belonging to the Period (*PER1*, *PER2*, *PER3*) and Cryptochrome (*CRY1*, *CRY2*) families, thus promoting their transcription [19]. In turn, PER and CRY proteins underpin a negative feedback mechanism: after translocating into the nucleus, they interact with the CLOCK:ARNTL complex and inhibit the transcription of their genes. This transcriptional repression results in a decrease in the levels of PER and CRY proteins, thus allowing the start of a new cycle [20,21].

A growing body of literature has been linking circadian misalignments to metabolic diseases. Sleep deprivation, night eating, and other unhealthy habits linked to circadian rhythm deregulation have negative consequences on weight gain and on the risk of developing weight-related pathologies [22,23]. Moreover, circadian rhythm disruption has been recently suggested to enhance inflammation and oxidative stress [24,25,26]. However, to date, the comprehensive mechanisms evoked by proinflammatory environmental factors on the epigenetic regulation of clock genes and the possible modifying role exerted by elevated BMI values remain largely unclear.

To investigate the potential role of overweight/obesity as a risk factor for circadian rhythm disruption in response to proinflammatory environmental triggers, we designed an epidemiological study investigating the effects of PM exposure on blood DNA methylation in a population of 200 women with an unhealthy body mass index (BMI), and therefore hypersusceptible due to their pre-existing, low-grade inflammatory status.

First, we evaluated whether exposure to airborne PM_10_ could alter the methylation of clock genes, potentially resulting in altered gene expression and circadian misalignments. In detail, we searched for an association between PM_10_ concentrations and the percentage of methylated CpGs in the promoter region of the clock genes *ARNTL*, *CLOCK*, *CRY1*, *CRY2*, *PER1*, *PER2*, and *PER3*, which play crucial roles in the regulation of the 24-h cellular rhythmicity. Then, we explored the role of the BMI as a variable that might influence the effect of PM on the methylation pattern of clock genes. Overall, this study provides new insights into the epigenetic outcomes of short-term PM exposure on the circadian *CLOCK* pathway in a population of hypersusceptible subjects.

## 2. Materials and Methods

### 2.1. Study Subjects

The present study was conducted on 200 women with overweight/obesity enrolled between 2010 and 2013 and randomly selected among the participants of the larger SPHERE study (ERC-2011-StG 282413), whose aim consists of investigating the molecular mechanisms evoked by PM exposure with a possible impact on human health [27]. All participants were residents in Lombardy at the time of enrollment, which was carried out at the Center for Obesity and Work (Department of Preventive Medicine, IRCCS Ca’ Granda—Ospedale Maggiore Policlinico) in Milan.

All subjects willing to participate in the study were asked to fill out a questionnaire to collect information about their eating habits and lifestyle, including current and past smoking habits, alcohol consumption, and physical activity. They were also required to indicate the place and date of birth, the residential address with characteristics of the house and traffic in the area, pathological and family history, educational qualifications, type and place of work.

Each candidate was subjected to measurements of weight, height, and abdominal circumference, and spirometry, electrocardiogram, and blood and urine tests were performed. BMI was calculated as the ratio between the subject’s weight (kg) and height squared (m^2^). According to the current guidelines [28], subjects who had a BMI between 25 and 29.9 kg/m^2^ were classified as overweight, whereas subjects with a BMI greater than or equal to 30 kg/m^2^ or higher were classified as obese.

Due to the importance of reducing bias associated with obesity, we used people-first language (and we encourage scientific authors to do so), according to the standard recommendation of European Association for the Study of Obesity (EASO), The Obesity Society (TOS), and Obesity Canada (OC) [29,30,31,32].

Patients with oncological, cardiac, neurodegenerative, or other chronic pathologies were excluded. The subjects fitting the inclusion criteria signed a written informed consent for the donation of blood samples, approved by the Ethics Committee of the Fondazione Ca’Granda—Ospedale Maggiore Policlinico. The blood withdrawal was carried out for all subjects in the morning (9.00–10.30 a.m.) and after overnight fasting to avoid introducing confounding factors linked to the physiological daily fluctuations in the methylation of the clock genes [33].

### 2.2. Assessment of PM Exposure

PM concentrations were recorded by the Regional Environmental Protection Agency (ARPA Lombardia) through monitoring stations located throughout Lombardy and available online as daily means. For each subject, daily PM concentrations were assigned considering the values registered by the nearest station to their home address. Using ArcGIS^®^ software (Esri), we geocoded each subject’s home address and each monitory station’s address. PM_10_ values were assigned according to each participant’s home address for the 6 days before blood sampling (from day −1 to day −6); instead, for the day of blood withdrawal (day 0) we considered the PM_10_ mean value registered by the three monitoring stations of Milan. Regarding PM_2.5_, as the presence of monitoring stations in the Lombardy area is very limited, we took into account the values registered in the city of Milan both for the day of blood sampling and the previous 6 days. In cases of incomplete series, missing values were attributed using an algorithm that integrated the annual average of the incomplete series and the PM concentrations of the nearest and more correlated monitors [34].

### 2.3. Sample Collection, DNA Extraction, and Bisulfite Treatment

Seven milliliters of whole blood were collected into EDTA tubes from each participant by venous phlebotomy. After centrifuging the blood tubes at 1200 g for 15 min to separate plasma, buffy coat, and erythrocytes, genomic DNA was extracted from the buffy coat fraction using the Wizard Genomic DNA Purification Kit (Promega; Madison, WI, USA) according to the manufacturer’s instructions. The concentration of the purified DNA was measured using the NanoDrop-1000 spectrophotometer (Thermo Fisher Scientific; Waltham, MA, USA). The DNA samples were plated at a concentration of 25 ng/µL in plates of 96 wells each and were treated with sodium bisulfite using the EZ-96 DNA Methylation-Gold™ Kit (Zymo Research; Irvine, CA, USA) following the manufacturer’s instructions. After elution, each DNA sample was divided into 10-µL aliquots using the Microlab STAR Automated Liquid Handling Workstation (Hamilton Company; Reno, NV, USA) and the plates were stored at −80 °C until use.

### 2.4. DNA Amplification and Pyrosequencing

Analysis of DNA methylation was performed via previously published methods with minor changes [15,35]. Briefly, 10 μL of bisulfite-treated template DNA was added with 25 μL of GoTaq Hot Start Green Master mix (Promega), 1 μL of forward primer (10 μM), and 1 μL of 5′ end-biotinylated reverse primer (10 μM) to set up a 50 μL PCR reaction. PCR cycling conditions and primer sequences are reported in Table 1.

The biotin molecule at the 5′ extremity of reverse primers was exploited to isolate a single DNA filament, which was subsequently used as a template for Pyrosequencing. The whole procedure was performed using the Pyromark^®^ Gold Q96 kit (QIAGEN GmbH, Hilden, Germany). Briefly, after incubating 15 μL of PCR product with Streptavidin Sepharose HP beads (Amersham BioSciences Ltd., Little Chalfont, UK), the biotin-labeled single-stranded DNA was purified, washed, denatured with 0.2 M NaOH, and washed again using the Pyrosequencing Vacuum Prep Tool (QIAGEN). After elution, the purified DNA filament was briefly incubated in an Annealing mix containing the sequencing primer (0.3 μM), and the plates were then heated up to 85 °C. Pyrosequencing was performed with the PyroMark MD System (QIAGEN). CpG sites were queried within the promoter regions of the following genes: *ARNTL*, *CLOCK*, *CRY1*, *CRY2*, *PER1*, *PER2*, *PER3*. 

The quantitative analysis of the methylation level at individual CpG positions within each gene’s promoter region was carried out using the Pyro Q-CpG software (Biotage, Uppsala, Sweden), which indicates the percentage of methylated cytosines out of the total number of cytosines (5-methyl-cytosine + unmethylated cytosines) at each CpG site of interest. Every sample was tested twice for each gene to guarantee the reproducibility of the experimental setting. Coefficients of variation for each assay are as follows: *ARNTL* = 0.2; *CLOCK* = 0.4; *CRY1* = 0.3; *CRY2* = 0.2; *PER1* = 0.2; *PER2* = 0.01; *PER3* = 0.02.

### 2.5. Statistical Analysis

Standard descriptive statistics were performed for all variables. Continuous variables were expressed as the mean ± standard deviation (SD) or as the median with first-, and third-quartile (Q1–Q3), as appropriate. Categorical data were reported as frequencies with percentages. 

To estimate the effect of PM_10_ and PM_2.5_ exposure on clock gene methylation (*CLOCK*, *ARNTL*, *CRY1*, *CRY2*, *PER1*, *PER2*, *PER3*) we applied linear mixed-effects models adjusted for age, BMI, smoking habits, percentage of lymphocytes, run, CpG site, season, temperature, and humidity. We adopted a repeated-measures design as DNA methylation measurements were run in duplicate for each subject, and the Pyrosequencing-based DNA methylation analysis tested a variable number of CpG positions according to CpG density in the promoter assay. We also included the effects of CpG position and run in the models to account for variation in methylation estimates due to experimental sources of variation. An unstructured covariance structure was used to model within-subject errors. The Kenward–Roger approximation was used to estimate the degrees of freedom in the denominator.

To examine the potential modifier role of the BMI on the association between PM exposure and clock genes, we added an interaction term between BMI and exposure in each model. To produce the estimates and plots of the PM-clock gene’s relationship at selected BMIs, we entered the levels of PM into the equation along with the range of values for each clock gene, at the mean value of continuous covariate and selected level for categorical variables. The cut-offs selected for BMI were 1st percentile (BMI = 25 kg/m^2^) and 99th percentile (BMI = 51 kg/m^2^). Estimated effects are reported as percentage changes and confidence intervals (CI) associated with an increase of 10 µg/m^3^ in each pollutant, which corresponded to (exp(β)-1) × 100.

Normality and linearity assumption by graphical inspection and the best model selection was based on the minimization of the Akaike information criterion and maximization of the explained variance of the model. A *p*-value of 0.05 was considered statistically significant. Statistical analyses were performed with SAS software (version 9.4; SAS, Cary, NC, USA) and R software (version 4.0.3; The R Foundation, Vienna, Austria).

## 3. Results

### 3.1. Characteristics of the Study Population, PM Assessment, and DNA Methylation

As summarized in Table 2, the study population included 200 women aged 52.7 ± 12.9 years, who were recruited as part of the SPHERE study [27]. According to their BMI, 55 subjects (27.5%) had overweight (25 ≤ BMI < 30), 72 (36.0%) had obesity Class I (30 ≤ BMI < 35) m and 73 (36.5%) had obesity Classes II and III (BMI ≥ 35). About half of the participants (45.5%) were current (13%) or former (32.5%) smokers.

The mean daily levels of the individual PM_10_ and PM_2.5_ values (expressed as μg/m^3^) attributed to each study subject and evaluated within 1 day to 7 days before the subject recruitment are reported in Table 3: PM_10_ averaged from 48.0 (2 days before recruitment) to 54.8 ng/m^3^ (4 and 13 days before recruitment).

The average methylation values for the clock genes ARNTL, CLOCK, CRY1, CRY2, PER1, PER2, and PER3 obtained by Bisulphite-Pyrosequencing are shown in Table 4. All the values indicated are expressed in terms of percentages of 5-methylcytosine (%5mC).

### 3.2. Association between PM and The Methylation of Clock Genes

We estimated the association between DNA methylation and exposure to PM_10_ measured on the day of blood collection (day 0), in the previous 6 days, and for the weekly average exposure. For each time lag, the association is expressed as a percent change in DNA methylation (% change) for 10 μg/m^3^ increments of PM_10_ (Supplementary Table S1). 

**Figure 1:**
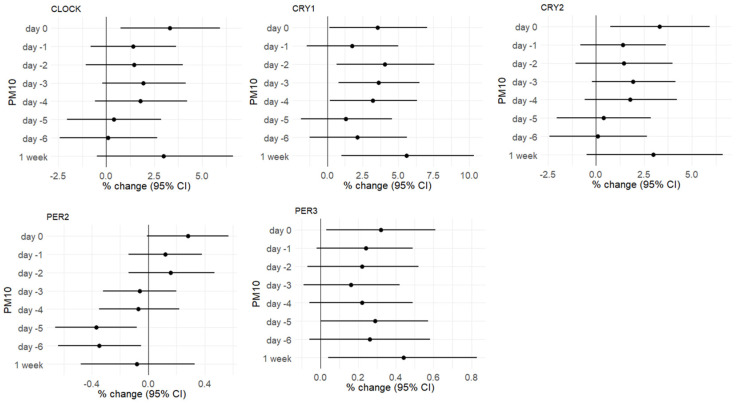
Plots showing the associations between short-term exposure to PM_10_ and the methylation of *CLOCK*, *CRY1*, *CRY2*, *PER2*, and *PER3* genes from day 0 to day −6 (single days and weekly average). Methylation values are provided as percentage changes (% changes) in methylation associated with 10-µg/m^3^ increments, estimated by multivariable regression models adjusted for age, BMI, smoking habits, percentage of lymphocytes, Pyrosequencing run, CpG site, season, temperature, and humidity.

As illustrated in Figure 1, we observed a negative association between PM_10_ exposure and CpG methylation for the *PER2* gene on days −5 and −6. Moreover, a significant positive association was observed for *CRY1* on days 0, −2, −3, and −4; for *CRY2* on day 0; for *CLOCK* on day −1 and for *PER3* on day −5. Considering the weekly mean PM_10_ exposure, a positive association was observed for *CRY1* and *PER3*. Conversely, the methylation status of CpG dinucleotides in the promoter region of *ARNTL* and *PER1* was not significantly associated with PM_10_ concentrations.

For the genes showing at least one significant association with a time lag, percentage changes (% changes) in methylation associated with 10-µg/m^3^ increments were reported.

**Figure 2:**
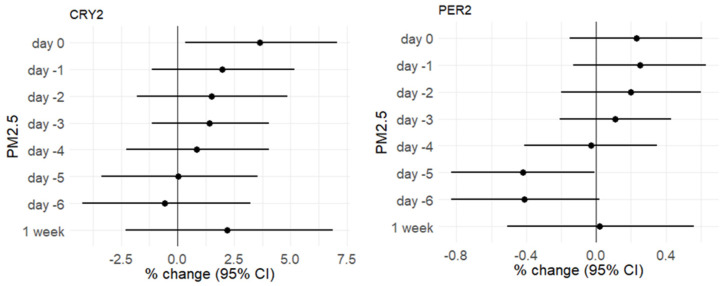
Plots showing the associations between short-term exposure to PM_10_ and the methylation of *CRY2* and *PER2* genes from day 0 to day −6. Methylation values are provided as percentage changes (% changes) in methylation associated with 10-µg/m^3^ increments, estimated by multivariable regression models adjusted for age, BMI, smoking habits, percentage of lymphocytes, Pyrosequencing run, CpG site, season, temperature, and humidity.

Regarding PM_2.5_, we observed that the methylation of *CRY2* was positively associated with PM_2.5_ increments on day 0, while the association was significantly negative for *PER2* on day −5 (Figure 2). Supplementary Table S2 reports the association coefficients for all the genes investigated.

### 3.3. Effect of Obesity on The Relationship between PM and Methylation

As obesity/overweight is an important susceptibility factor to the toxic effects of PM, we tested the possible role of BMI as an effect modifier of the association between the exposure to PM_10_ and PM_2.5_ and clock gene methylation (Supplementary Table S3). A significant effect modification of the BMI was observed for CRY2 (on days −5 and −6 for PM_10_; on days 0 and −5 for PM_2.5_), PER1 (for both PM_10_ and PM_2.5_, on days 0, −1, −2, −5, −6 and on the weekly average), and PER2 (on day −6 for PM_10_). Thus, Table 5 and Table 6 report the association between PM exposure (PM_10_ in Table 5 and PM_2.5_ in Table 6) and the methylation of the CRY2, PER1, and PER2 genes for selected BMI values (BMI = 25 and BMI = 51).

Interestingly, by applying this statistical model we observed PM-induced changes in DNA methylation for genes such as *PER1*, whose methylation level was not associated to PM exposure according to the previous model.

Considering the *p*-value for the study week, *PER1* is the only gene whose methylation change induced by both PM_10_ and PM_2.5_ is influenced by the BMI.

**Figure 3:**
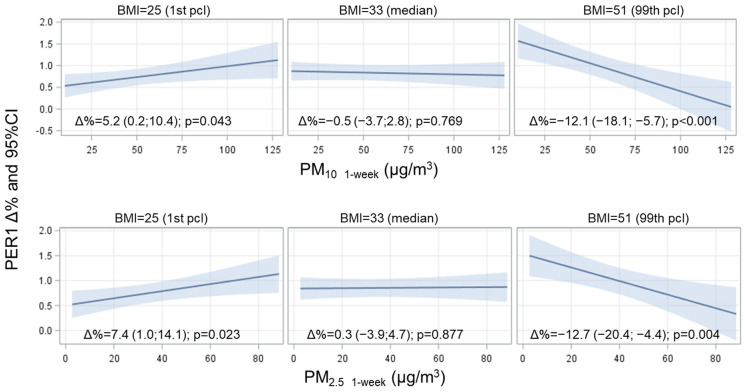
Interaction effect of PM and BMI on *PER1* methylation level. The strength of the association between PM and *PER1* at three selected levels of BMI (1st, 50th, and 99th percentile) is presented. Estimates were calculated from multivariate models adjusted for age, BMI, smoking habits, percentage of lymphocytes, Pyrosequencing run, CpG site, season, temperature, and humidity. Adjusted delta percent changes (Δ%) are reported for 10 µg/m^3^ increases in PM concentration. The *p*-values for the interaction term with BMI were *p* = 0.001 for PM_10_, and *p* < 0.001 for PM_2.5_. To produce the estimate, we entered the levels of PM into the equation along with the range of values for *PER1*, at the mean value of continuous covariate, and selected levels for categorical variables.

Figure 3 reports the effect of the BMI on the relationship between PM weekly averages and *PER1* methylation: for low BMI values (BMI = 25 kg/m^2^, 1st percentile) DNA methylation increases significantly as PM_2.5/10_ increases; instead, for high BMI values (BMI = 51 kg/m^2^, 99th percentile), a decrease in methylation is observed as PM_10_ increases. Median BMI values (BMI = 33 kg/m^2^) are also indicated and are not associated with significant changes in methylation.

## 4. Discussion

In the present study, conducted in a population of subjects with overweight/obesity, we observed an association between PM exposure measured in the week before the blood drawing and the methylation of circadian cycle genes (i.e., *CLOCK*, *CRY1*, *CRY2*, *PER1*, *PER2*, and *PER3*). In particular, we found that increments of PM_10_ concentrations were associated to the hypermethylation of the *CLOCK*, *CRY1*, *CRY2*, and *PER3* genes and to the hypomethylation of *PER2*. In contrast, *PER1* and *ARNTL* methylation levels appeared not to be correlated to PM_10_ levels. Regarding PM_2.5_, we reported an association with DNA methylation, which was positive for *CRY2* and negative for *PER2*.

Recently, it has been hypothesized that atmospheric particulate matter can affect the regulation of circadian rhythms, resulting in the desynchronization of both central and peripheral clocks [24,36]. However, the molecular mechanisms underlying the association between PM exposure and circadian cycle disruption have not been completely clarified.

A first potential mechanism could derive from the capacity of airborne particulate to stimulate the production of highly reactive free radicals and increase oxidative stress. Since CLOCK and ARNTL are sensitive to the intracellular redox state [37], this may justify the effect of a pro-oxidative agent such as PM on cellular circadian pathways. Accordingly, 24-h oscillations of H_2_O_2_ levels seem to have a modulatory effect on the activity of the CLOCK protein via cysteine oxidation [38]. Secondly, the fine and ultrafine PM may influence the epigenetic regulation of clock genes by stimulating the release of microvesicles by the lung epithelium and inflammatory cells [27]. Indeed, the SPHERE study demonstrated that the miRNA cargo of extracellular vesicles varies depending on PM concentrations, which could therefore regulate the levels of intracellular gene expression [39]. Additionally, the inflammatory response induced by PM could impinge upon the transcription of clock genes. In this regard, it has been demonstrated that the inducible transcription factor NF-κB, after being activated in response to inflammatory triggers, binds to the promoters of the *PER* and *CRY* clock repressors, thus inhibiting their expression [40]. This observation could be in agreement with our findings reporting that PM_10_ exposure associates with increased methylation of *CRY1*, *CRY2*, and *PER3*. Although there is mounting evidence on the capacity of PM to alter the expression of clock genes [41,42], the possible consequences of PM-induced methylation changes remain largely unclear. It has been reported that altered DNA methylation profiles in clock genes are often coupled to aberrant expression patterns, with possible implications in pathological conditions [43]. In addition, in the present work we only considered methylation occurring in promoter CpGs, while also non-CpG methylation of DNA can provide a mechanism for regulating gene expression by directly affecting the binding of transcription factors [44]. However, it is worth considering that DNA methylation is only one of the mechanisms that control the expression of circadian genes [45,46] and PM could modulate other molecular changes with a stronger impact on the regulation of clock genes rather than DNA methylation.

Moreover, to date, only a few studies have focused on PM-induced methylation changes occurring within the promoter region of circadian genes. To our knowledge, investigated methylation outcomes include cardiovascular disease [15], fetal development [18], and intervertebral disc degeneration [47]. Therefore, due to the gaps in the current knowledge, it is still difficult to speculate about the possible role of altered methylation patterns on circadian misalignments.

Our study was based on a population of women with overweight/obesity and it did not include any normal-weight subjects. Notably, the median value of the BMI in this population, corresponding to 33 kg/m^2^, is higher than the Italian average (25 kg/m^2^, referred to females) [48]. Hence, this difference between the study group and the overall population could hamper the generalization of our findings: indeed, the BMI is considered as an important determinant of hypersusceptibility when evaluating the biological mechanisms induced by exposure to particulate matter [49].

In this regard, our data indicate that the BMI significantly changes the association between PM exposure and the methylation of the *CRY2*, *PER1*, and *PER2* genes, suggesting that the BMI acts as an effect modifier. The modifying role of the BMI was particularly evident for *PER1*, especially because no significant methylation changes were observed in response to PM increases for this gene. More in detail, we reported that PM_2.5/10_ increments correspond to an increase in DNA methylation for low BMI values (BMI = 25, 1st percentile), while it markedly decreases in participants with severe obesity (BMI = 51, 99th percentile). To the best of our knowledge, *PER1* methylation levels have not been related to obesity or other metabolic alterations/disturbances. Although the overall methylation status of clock genes has been associated with glucose metabolism and dietary intake, no significant effect has been observed for DNA methylation changes in the promoter regions of individual genes, including *PER1* [50,51]. Moreover, the methylation level of *PER1* has been reported to be uncoupled to gene expression [52] and to be strongly sex-dependent [53,54], so this finding remains difficult to interpret.

Epigenetic mechanisms are strongly involved in the development of obesity and obesity-related health effects since altered methylation profiles of genes involved in inflammation, adipogenesis, and glucose metabolism have been described in individuals with overweight/obesity [51,55]. Considering that many aspects of cellular metabolism are under circadian control, it is likely that deregulating the expression of genes encoding key components of the core molecular clock machinery can have pronounced effects on both peripheral and central metabolic regulatory processes. As an example, lipid synthesis and accumulation are dependent on *ARNTL* expression levels in adipocytes [56].

DNA methylation signatures at circadian genes have been associated with obesity [51,57]; however, it is not clear whether these alterations are implicated in the etiology of obesity, or if they are a mere molecular consequence of other deregulated pathways. Interestingly, the methylation of clock genes has been found to change in response to dietary interventions [58,59], enforcing the hypothesis that energy metabolism and cellular rhythmicity are regulated reciprocally. In this complex interplay, it is conceivable that PM-induced DNA methylation changes occurring in the promoter region of clock genes might be modulated by the BMI, taken as an indicator of a healthy/unbalanced metabolic status.

The present study must be interpreted taking into account both strengths and limitations. First, due to the limited number of study subjects, it is possible that the associations observed were due to chance. In this regard, we applied a statistical model that took into account several potential confounders, all of which were considered as independent variables, to reduce bias and chance findings. However, the findings need to be replicated in larger groups of subjects, possibly including subjects with a BMI < 25 and males, in order to allow generalizability of the findings. Another possible limitation concerns the attribution of PM_2.5_ concentrations to the study population. Since the number of available PM_2.5_-recording monitoring stations was much lower than the number of PM_10_ monitoring stations, the PM_2.5_ values assigned to the participants may not fully mirror the real concentrations experienced by study subjects. However, since the data obtained are generally in agreement with those relative to PM_10_ (of which a large percentage is made of particles with an aerodynamic diameter <2.5 µm in the study area), it is unlikely that this could affect the interpretation of our results.

In the future, it will be useful to assess whether the association between PM and the methylation of circadian genes exist also in normal-weight individuals and, since PM fuels inflammation, whether there is an association between the methylation of clock genes and inflammatory disease development. Furthermore, it would be interesting to evaluate the effect exerted by other components of air pollution, such as ozone, nitrogen, and carbon oxides, as they could be implicated in modulating the detected changes in DNA methylation.

## 5. Conclusions

This study highlights the association between the exposure to atmospheric particulate matter and clock gene methylation. In addition, our data suggest that BMI could be a susceptibility factor capable of altering the effect of PM on the considered DNA methylation outcomes. Further studies will be necessary to unravel a possible impact of methylation changes on the expression of clock genes and the regulation of circadian rhythmicity, as well as the role of obesity as a potential risk factor conferring hypersensitivity to PM.

## Figures and Tables

**Table 1:** Pyrosequencing assay information.

Gene	Chromosome Position ^1^	CpG Sites	Primers: Forward (F) Reverse (R) Sequencing (S)		Sequencing Length	T° Annealing
***ARNTL***	chr11:13275818 -13278227	2	F R S	GGGGTTAGTTTGGGTAATAGAATTAG Bio-TAAACTCCCTAAATAAAAAAACAAC TTATTTTATTTTATTTTAGT	38 bp	54 °C
***CLOCK***	chr4:55547142 -55547530	2	F R S	TTTTTAGGAGATGGGAGAAGATGT Bio-TAAAAAATCCAAAAACCAAAAAAAA TTTTTTGTTAATATT	28 bp	51.5 °C
***CRY1***	chr12:105617622 -105618592	3	F R S	TTTGTGAGGGAAGGTTTAGTTT Bio-AACAATTTCCAAACCCTCC TTTTTAAGGGTTATGAG	27 bp	56 °C
***CRY2***	chr11:45846906 -45847578	4	F R S	TGTTTTTTGAGATTTGGTTTATTTT Bio- CCAAAACCCCTCTACCATTAACTA TGTTTTTTGAGATTTGGTTTATTTT	33 bp	54 °C
***PER1***	chr17:8151724 -8152661	3	F R S	TAGGGTTAGGGATTGGAGAATAGA Bio-ACCCAAACAAAAAACACACTATC GGGTTAGGAGTGTAGATTTT	27 bp	52 °C
***PER2***	chr2:238288036 -238291073	3	F R S	TGAGAAAGGTAGTATTTTTAAGG Bio-AAAACTCCACATACCCCACAC AGGAGGTTGTTTTGGGAGAT	34 bp	52 °C
***PER3***	chr1:7784068 -7785195	3	F R S	TGTTTGTTATTGATTGTAAAGTGAG Bio-AATTTAAATCCCCCTTTCCCTAC TGTTTGTTATTGATTGTAAAGTGAG	25 bp	52 °C

^1^ As reported by the UCSC Genome Browser, GRCh38/hg38 assembly.

**Table 2:** Characteristics of the study participants.

Characteristics		Value
Age (years ± SD)		52.7 ± 12.9
BMI (kg/m^2^ ± SD)		33.8 ± 5.5
Categorical BMI (number of subjects (%))		
	25 ≤ BMI < 30 (Overweight)	55 (27.5%)
	30 ≤ BMI < 35 (Obesity Class I)	72 (36.0%)
	BMI ≥ 35 (Obesity Classes II and III)	73 (36.5%)
Smoking habits (number of subjects (%))		
	Nonsmoker	109 (54.5%)
	Ex-smoker	65 (32.5%)
	Current smoker	26 (13%)
Percentage of lymphocytes (mean ± SD)		30.9% ± 7.2%
Season of enrollment (number of subjects (%))		
	Winter	57 (28.5%)
	Spring	55 (27.5%)
	Summer	28 (14.0%)
	Autumn	60 (30.0%)
Temperature (°C ±SD)		69.8 ± 14.7
Humidity (% ±SD)		12.7 ± 7.6

**Table 3:** PM concentrations recorded by ARPA Lombardia monitoring stations.

PM Size	Days before Blood Sampling	Mean (μg/m^3^)	SD	First Quartile (Q1)	Median (Q2)	Third Quartile (Q3)
PM_10_	Day 0	47.2	28.8	25.7	37.3	62.2
	Day −1	41.8	30.2	22.0	31.0	56.0
	Day −2	39.8	27.2	21.0	31.0	52.0
	Day −3	41.3	29.9	22.0	33.0	56.0
	Day −4	43.6	28.4	23.0	36.0	60.0
	Day −5	43.6	27.5	24.0	36.0	59.0
	Day −6	42.9	26.7	24.0	36.0	53.0
	Weekly mean	42.8	22.9	26.7	36.4	54.0
PM_2.5_	Day 0	32.9	23.5	16.0	25.8	45.0
	Day −1	30.9	22.6	14.0	24.0	43.0
	Day −2	30.4	22.6	14.5	25.0	38.0
	Day −3	32.2	26.5	13.0	25.0	44.0
	Day −4	32.9	23.8	16.0	25.0	46.0
	Day −5	31.2	21.0	14.3	25.3	41.5
	Day −6	30.8	20.0	16.0	25.0	43.0
	Weekly mean	31.3	18.9	16.0	27.9	40.6

**Table 4:** Methylation values of clock genes.

Gene	Mean (% mCpG)	SD	First Quartile (Q1)	Median (Q2)	Third Quartile (Q3)	Min	Max
***ARNTL***	1.1	0.7	1.1	0.8	1.3	0	7.2
***CLOCK***	1.9	1.6	1.2	0.8	2.7	0.3	7.5
***CRY1***	2	1.5	1.7	1.1	2.5	0	10.3
***CRY2***	1.2	0.5	1.2	1	1.4	0	3.7
***PER1***	1.5	1	1.4	0.8	2.2	0	5.3
***PER2***	78.7	3.5	79	76.9	80.8	60.5	86.7
***PER3***	84.9	3.7	85.5	82.5	87.4	73.2	93.3

**Table 5:** Association between PM_10_ exposure and the methylation of the *CRY2*, *PER1*, and *PER2* genes for selected BMI values. Significant associations (*p*-value < 0.05) are highlighted in bold characters.

Methylation Genes Δ% (95% CI) *p*−Value	CRY2		PER1		PER2	
	BMI = 25	BMI = 51	BMI = 25	BMI = 51	BMI = 25	BMI = 51
**PM10 Exposure**						
**Day 0**	5.4 (1.5; 9.4)	−1.2 (−6.8; 4.7)	6.9 (3.3; 10.7)	−9.2 (−13.9; −4.2)	0.5 (0.1; 1)	−0.2 (−0.9; 0.5)
	0.007	0.673	<0.001	<0.001	0.020	0.521
**Day −1**	3.2 (−0.1; 6.6)	−2 (−6.8; 3)	4.7 (1.2; 8.3)	−8.4 (−12.5; −4.1)	0.2 (−0.2; 0.6)	0 (−0.6; 0.6)
	0.058	0.424	0.009	0.000	0.370	0.958
**Day −2**	3.2 (−0.9; 7.5)	−1.2 (−6.5; 4.4)	2.7 (−1.4; 7)	−6.1 (−11; −1)	0.5 (0; 1)	−0.3 (−1; 0.3)
	0.129	0.665	0.192	0.021	0.059	0.352
**Day −3**	1.8 (−2; 5.8)	2.6 (−4.5; 10.2)	1.2 (−2.7; 5.2)	−4.2 (−10.7; 2.7)	0.2 (−0.3; 0.7)	−0.5 (−1.3; 0.4)
	0.351	0.481	0.557	0.224	0.470	0.266
**Day −4**	2 (−2.2; 6.4)	1.5 (−5.3; 8.8)	0.6 (−3.5; 5)	−1.8 (−8.2; 5.1)	0.2 (−0.3; 0.8)	−0.6 (−1.4; 0.2)
	0.352	0.676	0.765	0.598	0.349	0.163
**Day −5**	4 (−0.3; 8.6)	−5.3 (−11.1; 0.8)	3.8 (−0.3; 8)	−10.5 (−15.6; −5.1)	0 (−0.5; 0.5)	−0.9 (−1.7; −0.2)
	*0.068*	*0.089*	*0.070*	*<0.001*	*0.985*	*0.011*
**Day −6**	3.1 (−0.9; 7.2)	−4.4 (−9.4; 1)	4.3 (0.5; 8.3)	−9.6 (−14; −5)	0 (−0.4; 0.5)	−0.9 (−1.5; −0.2)
	*0.134*	*0.110*	*0.026*	*<0.001*	*0.842*	*0.009*
**1 week**	6.2 (0.9; 11.7)	−2.5 (−9.6; 5.2)	5.2 (0.2; 10.4)	−12.1 (−18.1; −5.7)	0.3 (−0.3; 0.9)	−0.7 (−1.6; 0.1)
	0.021	0.506	0.043	<0.001	0.318	0.100

**Table 6:** Association between PM_2.5_ exposure and the methylation of the *CRY2*, *PER1*, and *PER2* genes for selected BMI values. Significant associations (*p*-value < 0.05) are highlighted in bold characters.

Methylation Genes Δ% (95% CI) *p*-Value	CRY2		PER1		PER2	
	BMI = 25	BMI = 51	BMI = 25	BMI = 51	BMI = 25	BMI = 51
**PM10 exposure**						
**Day 0**	8 (3; 13.2)	−4.4 (−11.6; 3.4)	9.7 (4.8; 14.8)	−10.1 (−16.6; −3)	0.7 (0.1; 1.2)	−0.7 (−1.6; 0.2)
	0.002	0.258	<0.001	0.006	0.019	0.144
**Day −1**	2.9 (−1.2; 7.2)	0.1 (−6.4; 7.1)	5.8 (0.9; 10.8)	−8.3 (−14.5; −1.7)	0.4 (−0.1; 0.9)	0 (−0.9; 0.8)
	0.168	0.969	0.019	0.014	0.100	0.932
**Day −2**	3.1 (−1.9; 8.4)	−2.3 (−9.6; 5.4)	3.9 (−1.3; 9.3)	−8.5 (−15; −1.5)	0.5 (−0.1; 1.1)	−0.5 (−1.4; 0.4)
	0.221	0.542	0.141	0.019	0.103	0.303
**Day −3**	2.6 (−1.5; 7)	−1.2 (−8.9; 7.2)	2.3 (−1.9; 6.8)	−5 (−12.3; 3)	0.5 (−0.1; 1)	−0.7 (−1.7; 0.3)
	0.217	0.768	0.288	0.215	0.080	0.167
**Day −4**	1.3 (−4.2; 7)	−1 (−9.1; 7.7)	1.5 (−4; 7.2)	−0.3 (−8.1; 8.2)	0.3 (−0.3; 1)	−0.7 (−1.7; 0.3)
	0.656	0.813	0.604	0.941	0.334	0.170
**Day −5**	4.2 (−1.5; 10.2)	−8.7 (−16.3; −0.5)	4.6 (−0.7; 10.3)	−11.9 (−18.7; −4.5)	−0.1 (−0.7; 0.6)	−1.2 (−2.2; −0.2)
	0.148	0.038	0.090	0.002	0.828	0.018
**Day −6**	2.8 (−2.8; 8.7)	−6.5 (−13.3; 0.8)	6.3 (0.8; 12.2)	−11.3 (−17.2; −5)	0 (−0.7; 0.6)	−1 (−1.9; −0.2)
	0.327	0.079	0.026	0.001	0.966	0.021
**1 week**	5.9 (−0.6; 12.7)	−4.6 (−13.4; 5)	7.4 (1; 14.1)	−12.7 (−20.4; −4.4)	0.5 (−0.2; 1.3)	−0.9 (−2; 0.2)
	0.074	0.332	0.023	0.004	0.184	0.115

## Data Availability

The data presented in this study is available upon request to the corresponding author.

## Supplementary Materials

The following are available online at https://www.mdpi.com/1660-4601/18/3/1122/s1, Table S1: Associations between short-term exposure to PM_10_ and clock methylation genes, Table S2: Associations between short-term exposure to PM_2.5_ and clock methylation genes, Table S3: Significance of the interaction term testing the modifier role of BMI, on the association between PM exposure and clock methylation genes.
