# Supplementary material for: Effects of PM Exposure on the Methylation of Clock Genes in A Population of Subjects with Overweight or Obesity
Source: Int J Environ Res Public Health. 2021 Jan 27;18(3):1122. doi: 10.3390/ijerph18031122 (PMC7908270; doi:10.3390/ijerph18031122)
Supplement: Supplementary file 1 [file ijerph-18-01122-s001.pdf]

Table S1. Associations between short-term exposure to PM_{10} and clock methylation genes.

**Methylation Genes** **Δ% (95% CI)** ***p-Value***	**CLOCK**	**ARNTL**	**CRY1**	**CRY2**	**PER1**	**PER2**	**PER3**
PM10 exposure							
Day 0	2.16 (−1.86; 6.34)	0.19 (−1.8; 2.23)	3.54 (0.15; 7.04)	3.31 (0.74; 5.94)	0.92 (−1.46; 3.36)	0.28 (−0.01; 0.57)	0.32 (0.03; 0.61)
	0.295	0.849	**0.040**	**0.012**	0.450	0.056	**0.032**
Day 1	3.9 (0.3; 7.63)	−0.48 (−2.19; 1.27)	1.74 (−1.44; 5.02)	1.39 (−0.81; 3.64)	−0.65 (−2.9; 1.66)	0.12 (−0.14; 0.38)	0.24 (−0.02; 0.49)
	**0.034**	0.589	0.285	0.216	0.577	0.366	0.068
Day 2	3 (−1.14; 7.32)	−0.79 (−2.75; 1.21)	4.04 (0.65; 7.54)	1.44 (−1.06; 4)	−0.97 (−3.38; 1.5)	0.16 (−0.14; 0.47)	0.22 (−0.07; 0.52)
	0.157	0.435	**0.019**	0.261	0.436	0.298	0.134
Day 3	0.91 (−2.61; 4.56)	−0.94 (−2.55; 0.7)	3.61 (0.8; 6.51)	1.94 (−0.22; 4.14)	−0.75 (−2.81; 1.35)	−0.06 (−0.32; 0.2)	0.16 (−0.09; 0.42)
	0.615	0.259	**0.012**	0.078	0.477	0.654	0.213
Day 4	0.69 (−3.2; 4.73)	−1.12 (−2.93; 0.73)	3.21 (0.18; 6.33)	1.78 (−0.58; 4.21)	−0.36 (−2.69; 2.01)	−0.07 (−0.35; 0.22)	0.22 (−0.06; 0.49)
	0.733	0.233	**0.038**	0.140	0.760	0.644	0.118
Day 5	3.12 (−0.94; 7.34)	−1.41 (−3.32; 0.54)	1.31 (−1.86; 4.58)	0.38 (−2.04; 2.85)	−1.82 (−4.08; 0.5)	−0.37 (−0.66; −0.08)	0.29 (0; 0.57)
	0.133	0.154	0.420	0.761	0.122	**0.012**	**0.047**
Day 6	1.51 (−2.7; 5.91)	0.17 (−1.86; 2.24)	2.13 (−1.25; 5.62)	0.09 (−2.43; 2.67)	−1.14 (−3.53; 1.3)	−0.35 (−0.64; −0.05)	0.26 (−0.06; 0.58)
	0.486	0.873	0.219	0.946	0.355	**0.023**	0.108
1 week	4 (−1.6; 9.92)	−1.33 (−3.99; 1.4)	5.57 (0.99; 10.35)	2.99 (−0.5; 6.62)	−1.58 (−4.82; 1.76)	−0.08 (−0.48; 0.33)	0.44 (0.04; 0.83)
	0.164	0.334	**0.017**	0.094	0.347	0.714	**0.030**

Estimates are provided as percentage changes in methylation associated with 10-μg/m^3 PM_{10} increment, estimated by multivariable regression models adjusted for age, BMI, smoking habits, percentage of lymphocytes, run, CpG site, season, temperature, and humidity.

Table S2. Associations between short-term exposure to $PM_{2.5}$ and clock methylation genes.

**Methylation Genes** **Δ% (95% CI)** ***p-Value***	**CLOCK**	**ARNTL**	**CRY1**	**CRY2**	**PER1**	**PER2**	**PER3**
PM2.5 exposure							
Day 0	−1.83 (−6.95; 3.56)	0.16 (−2.46; 2.84)	3.69 (−0.93; 8.53)	3.65 (0.33; 7.07)	2.56 (−0.68; 5.89)	0.23 (−0.15; 0.61)	0.05 (−0.33; 0.43)
	0.496	0.908	0.119	**0.031**	0.121	0.234	0.793
Day 1	0.15 (−5.14; 5.74)	0.2 (−2.43; 2.89)	0.42 (−3.97; 5.02)	1.97 (−1.13; 5.17)	0.69 (−2.59; 4.09)	0.25 (−0.13; 0.63)	0.11 (−0.28; 0.51)
	0.956	0.884	0.853	0.213	0.682	0.192	0.573
Day 2	−0.06 (−5.4; 5.58)	−0.57 (−3.25; 2.18)	1.89 (−2.71; 6.71)	1.49 (−1.79; 4.87)	−0.85 (−4.11; 2.52)	0.2 (−0.2; 0.6)	0.06 (−0.33; 0.46)
	0.982	0.678	0.424	0.374	0.615	0.334	0.759
Day 3	−1.19 (−5.45; 3.26)	−0.54 (−2.55; 1.51)	2.51 (−0.95; 6.09)	1.42 (−1.15; 4.04)	−0.1 (−2.67; 2.53)	0.11 (−0.21; 0.43)	0.1 (−0.21; 0.41)
	0.591	0.602	0.156	0.279	0.937	0.510	0.540
Day 4	−2.16 (−7.16; 3.11)	−0.7 (−3.13; 1.78)	1.62 (−2.38; 5.79)	0.85 (−2.27; 4.06)	0.51 (−2.55; 3.68)	−0.03 (−0.41; 0.35)	0.04 (−0.32; 0.41)
	0.412	0.573	0.430	0.596	0.744	0.872	0.812
Day 5	0.22 (−5.35; 6.12)	−1.31 (−3.93; 1.38)	−1.16 (−5.46; 3.35)	0.03 (−3.37; 3.54)	−1.6 (−4.72; 1.63)	−0.42 (−0.83; −0.01)	0.04 (−0.36; 0.44)
	0.939	0.335	0.607	0.988	0.326	**0.042**	0.847
Day 6	−0.9 (−6.84; 5.42)	0.89 (−1.93; 3.79)	−3.57 (−8.17; 1.25)	−0.57 (−4.22; 3.23)	−1.3 (−4.78; 2.31)	−0.41 (−0.83; 0.02)	0.03 (−0.4; 0.47)
	0.774	0.540	0.143	0.765	0.472	0.064	0.886
1 week	−2.09 (−9.02; 5.37)	−0.63 (−4.15; 3.02)	2.17 (−3.79; 8.49)	2.18 (−2.29; 6.86)	−0.14 (−4.47; 4.38)	0.02 (−0.51; 0.56)	0.09 (−0.43; 0.61)
	0.571	0.730	0.481	0.342	0.949	0.940	0.735

Estimates are provided as percentage changes in methylation associated with 10-μg/m^3 $PM_{2.5}$ increment, estimated by multivariable regression models adjusted for age, BMI, smoking habits, percentage of lymphocytes, run, CpG site, season, temperature, and humidity.

Table S3. Significance of the interaction term testing the modifier role of BMI, on the association between PM exposure and clock methylation genes.

**Interaction Term *p*-Value (PM Exposure)*BMI**	**CLOCK**	**ARNTL**	**CRY1**	**CRY2**	**PER1**	**PER2**	**PER3**
PM10 exposure							
Day 0	0.342	0.620	0.356	0.126	**<0.001**	0.142	0.307
Day 1	0.385	0.693	0.488	0.160	**0.000**	0.773	0.905
Day 2	0.645	0.961	0.364	0.293	**0.028**	0.113	0.485
Day 3	0.919	0.758	0.826	0.880	0.288	0.318	0.574
Day 4	0.593	0.564	0.964	0.938	0.650	0.207	0.373
Day 5	0.973	0.140	0.207	**0.030**	**0.001**	0.078	0.401
Day 6	0.085	0.638	0.067	**0.036**	**<0.001**	**0.032**	0.678
1 week	0.482	0.582	0.245	0.110	**<0.001**	0.097	0.406
PM2.5 exposure							
Day 0	0.287	0.435	0.258	**0.044**	**<0.001**	0.066	0.192
Day 1	0.567	0.689	0.854	0.659	**0.004**	0.496	0.272
Day 2	0.563	0.851	0.478	0.378	**0.013**	0.140	0.571
Day 3	0.994	0.783	0.932	0.675	0.176	0.147	0.274
Day 4	0.582	0.391	0.956	0.924	0.835	0.228	0.202
Day 5	0.923	0.120	0.638	**0.044**	**0.005**	0.160	0.454
Day 6	0.259	0.663	0.334	0.068	**0.001**	0.086	0.526
1 week	0.554	0.409	0.478	0.114	**0.001**	0.069	0.266

p-value from multivariate linear regression model adjusted for age, BMI, the interaction between PM exposure and BMI, smoking habits, percentage of lymphocytes, run, CpG site, season, temperature, and humidity.
